# Oil-Based Fungal Pigment from *Scytalidium cuboideum* as a Textile Dye

**DOI:** 10.3390/jof6020053

**Published:** 2020-04-22

**Authors:** Mardonio E. Palomino Agurto, Sarath M. Vega Gutierrez, R. C. Van Court, Hsiou-Lien Chen, Seri C. Robinson

**Affiliations:** 1 Department of Wood Science and Engineering, Oregon State University, Corvallis, OR 97331, USA; mardonio.palomino@oregonstate.edu (M.E.P.A.); sarathth@yahoo.co.uk (S.M.V.G.); ray.vancourt@oregonstate.edu (R.C.V.C.); 2 College of Business, Oregon State University, Corvallis, OR 97331, USA; hsiou-lien.chen@oregonstate.edu

**Keywords:** fungal pigment, natural dye, spalting, *Scytalidium cuboideum*, dramada, sustainable clothing

## Abstract

Identification of effective natural dyes with the potential for low environmental impact has been a recent focus of the textile industry. Pigments derived from spalting fungi have previously shown promise as textile dyes; however, their use has required numerous organic solvents with human health implications. This research explored the possibility of using linseed oil as a carrier for the pigment from *Scytalidium cuboideum* as a textile dye. Colored linseed oil effectively dyed a range of fabrics, with natural fibers showing better coloration. Scanning electron microscopy (SEM) revealed a pigment film over the fabric surface. While mechanical testing showed no strength loss in treated fabric, colorfastness tests showed significant changes in color in response to laundering and bleach exposure with variable effects across fabric varieties. SEM investigation confirmed differences in pigmented oil layer loss and showed variation in pigment crystal formation between fabric varieties. Heating of the pigmented oil layer was found to result in a bright, shiny fabric surface, which may have potential for naturally weatherproof garments.

## 1. Introduction

Natural dyes and pigments have a long history of use for coloring textiles, from ancient Egypt to the oldest South American cultures [1,2,3,4,5]. Artificial dyes currently dominate the market due to their ease of mass production, low price, and color variety [5]. However, most modern textile colorants are produced using hazardous chemicals and many contribute to water pollution through the production of effluents [6,7]. In recent decades, the low environmental impact and sustainability of natural dyes have become more desired by consumers, driving a shift in the market back to natural sources of coloration [8,9].

There are presently a number of natural colorant alternatives available, such as those derived from barks, fungal pigments, insects, and minerals [3]. Most have never been commercialized due to concerns about their sustainability, cost effectiveness, and profitability [10]. In addition, natural dyes often have problems with colorfastness, and, while methods have been developed to improve dye uptake [11,12,13], they are energy intensive and increase the production price [14,15,16].

**Figure 1:**
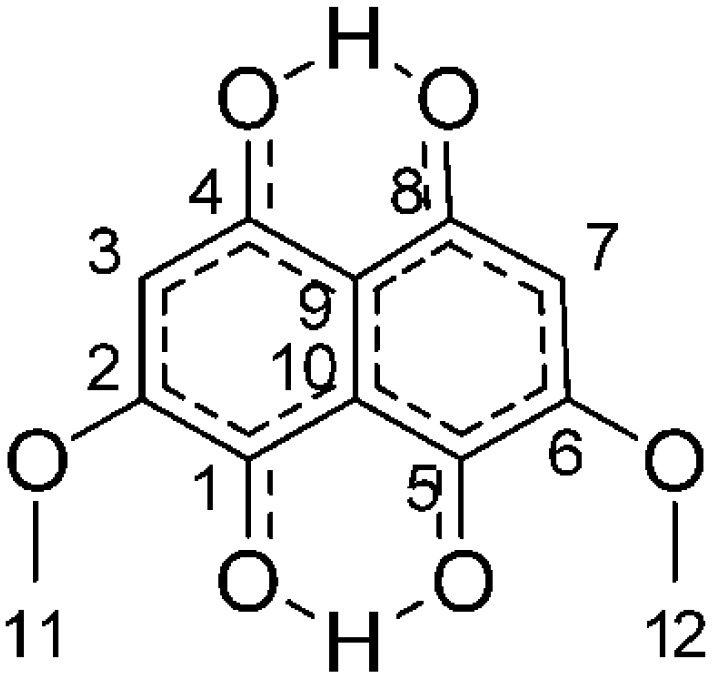
The structure of dramada, the orange and red crystal pigment from *Scytalidium cuboideum.*

One source of natural coloration that has been suggested as a replacement for synthetic dyes in fabric dyeing are pigments extracted from wood-rotting fungi. Research by Weber et al. [17] has shown that fungal pigments, when carried in dichloromethane (DCM), with and without mordants, showed promise for dyeing fabrics, using a dripping process. The pigments from four fungi were investigated: *Chlorociboria aeruginosa* (Oeder), Seaver, and *C. aeruginascens* (Nyl.) Kanouse, which produces a blue-green pigment known as xylindein, *Scytalidium cuboideum* (Sacc. & Ellis) Sigler & Kang, which produces a red pigment known as draconin red (Figure 1) that exists as both orange and red crystals, and *Scytalidium ganodermophthorum* Kang, Sigler, Y.W. Lee & S.H. Yun, which produces an unnamed yellow pigment. Further studies showed that polyester absorbed more pigment than other synthetic fabrics and that these colors were stable over time [17,18]. Hinsch [19] later confirmed that *S. cuboideum* and *C. aeruginosa* could be used to dye textiles with no mordant and attributed this to the carbon–carbon interaction of the main quinone structures.

Despite the success of early testing, the fungal pigments mentioned above never gained commercial traction. This is likely due to the need for the pigments to be carried in DCM, which is a potential human carcinogen and a known greenhouse gas [20,21,22]. Other solvents were tested for their ability to extract the pigments, and while tetrahydrofuran (THF), acetonitrile (ACN), and acetone were all moderately capable of extraction, all three interacted with the pigments and caused color change [23]. Due to these issues, an entirely new type of solvent was explored: natural oils. These oils proved to be highly successful at carrying the fungal pigments, though not at extracting them, with raw linseed oil allowing for the highest pigment stability of those tested [24]. However, of the tested pigments, only the red pigment from *S. cuboideum* was found to effectively color fabric, although it showed variation in coloration based on the material to which it was applied [25].

The purpose of study was to compare the dyeing capability, heat stability (in different washing temperatures), and pH stability (washing in different detergents), of three soft-rot spalting fungi, carried in raw linseed oil, on various fabrics. While it is already known that the pigments from these fungi are reasonable textile dyes when carried in DCM [17,18], this carrier is not practical for commercial scale use due to toxicity issues. An alternative carrier proposed is raw linseed oil, which is known to carry the pigments at high concentrations but has never been tested in textile applications. Using the oil carrier would result in a dyeing process with fewer environmental issues than the DCM process. Analysis via scanning electron microscopy (SEM) will further the understanding of the interaction between the fungal pigments and the fabrics. The research will assess if the oil changes the structure of the pigments, how the pigments bind to textiles, and if the reaction of the pigments changes due to temperature and pH changes based upon the oil carrier. 

This work prepares the fungal pigments to fully enter into the commercial dye industry by assessing the viability of a more eco-friendly carrier and removing the largest hurdle currently facing the soft-rot fungal pigments, their DCM requirement for application.

## 2. Materials and Methods

All testing methodology followed either accepted the American Society of Testing Material (ASTM) standards (such as for fabric testing) or were based on testing used in previous work with these spalting pigments on textiles (see citations below).

### 2.1. Fungal Growth and Pigment Extraction

Cultures of *S. cuboideum* (UAMH 4802, isolated from oak lumber, location unknown) were used to inoculate malt agar plates amended with white rotted wood chips, following the procedure outlined in Robinson et al. [23]. Plates were allowed to grow for 2–3 weeks before they were dried for 24 h in a fume hood, extracted into DCM, and standardized to CIE L*a*b* values of *L* = 82.32, *a* = 26.84, and *b* = 13.19, as described in Robinson et al. [23]. After the standardization, raw linseed oil (Sunnyside) was mixed with the pigment carried in DCM and placed on a stir plate for 48 h in order to evaporate the solvent and leave the pigment suspended in the oil, following the methodology by Palomino Agurto et al. [25]. This pigmented oil was used for further testing.

### 2.2. Fabrics Tested

Unfinished 100% fabrics of polyester, nylon, cotton, and wool were used (see Table S1). Per each type of fabric, two different densities were used: low-density and high-density. To establish a difference between low-density and high-density, a minimum difference of 40 yarns per inch in the count number was established. Low-density nylon was discarded because its finishing process did not allow dying by the pigmented oil.

### 2.3. Mechanical Testing

Mechanical testing was done to evaluate the impact of the oil-pigments on tensile and tear strength of the fabrics, with samples for mechanical testing prepared according to the American Society of Testing Material (ASTM 2013). Submersion dying was carried out using borosilicate glass beakers (250 mL) (brand VWR, Randor, PA, USA), filled with 100 mL of pigmented oil from *S. cuboideum*. Samples from tested fabrics were separately submerged in the pigment solution in the uncovered beakers for 1 h. Once the time of exposure was achieved, samples were removed from the borosilicate beakers and placed over a metal mesh to dry for 48 h. 

#### 2.3.1. Tear Strength Test

The tear strength test was performed according to the ASTM D1424 using a falling-pendulum digital Elmendorf-type (produced by SDL ATLAS, Rock Hills, SC, USA) to measure the force required to propagate a single-rip tear. This test was performed on the samples of the four fabrics in filling directions. Twelve repetitions were performed on both dyed and undyed control samples (treated with only raw linseed oil), making a total of 36 samples per fiber fabric and 144 samples in total.

Estimated least squares means tests with Tukey adjustment was performed using SAS 9.8 (SAS Institute, Cary, NC, USA) to compare the effect of treatment and fabric type on stress (MPa) or force (N). The least squares means for main effects were reported if the main effect was statistically significant and not part of any statistically significant interaction or when the main effect was statistically significant and the effect of that factor was always in the same direction for the interacting variable(s). Selected samples were later evaluated using SEM analysis (below) to assess the behavior of pigment crystals inside the fabric structures.

#### 2.3.2. Tensile Strength Test

The breaking (tensile strength) test was performed according to the ASTM D5034-09 (2013), using the Universal Testing Machine Instron Model 5582 (produced by Instron Company, Norwood, MA, USA). Samples were loaded into the tool and then exposed to tensile forces until breaking occurred. The test was repeated for 30 samples for each fabric/density type, with 10 controls, 10 samples dyed with only raw linseed oil, and 10 samples dyed with pigmented oil. After the tensile strength test, affected samples were analyzed using SEM to evaluate any physical change in the attachment of the pigment to the fibers.

Estimated least squares means tests with Tukey adjustment were performed using SAS 9.8 (SAS Institute, Cary) to compare the effect of treatment and fabric type on f (N), and least squares means for main effects were reported if the main effect was statistically significant and not part of any statistically significant interaction or when the main effect was statistically significant and the effect of that factor was always in the same direction for the interacting variable(s). Selected samples were later evaluated using SEM analysis (below) to assess the behavior of pigment crystals inside the fabric structures.

### 2.4. Analysis of Colorfastness

#### 2.4.1. Color Variation Across Fabrics

Difference in pigment uptake and coloration were compared between fabrics. Fabric samples were cut into 5.08 × 5.08 cm, and dripping was used to apply fungal pigment. This consisted of applying pigment suspended in raw linseed oil onto the surface of each fabric sample using a disposable pipette. Fifteen drops of the oil-solubilized pigments were applied onto each fabric sample using a 1 mL pipette (brand Gilson, Lewis Center, OH, USA), with drops weighing an average 0.02443 g.

Control samples were compared to samples colored using the dripping methodology, and color analysis was performed on the samples using a Konica Minolta Chroma Meter CR-5 colorimeter, using the CIE L*a*b* color space, with the ∆E calculation 2000. A one-way ANOVA was performed to test for the effect of fabric type on the response of color variation (∆E) using SAS 9.8 (SAS Institute, Cary).

#### 2.4.2. Laundry Test

Colorfastness to laundering with and without bleach was performed on the fabric samples dyed with the dripping method. The washing test was performed according to the AATCC method 61-2013 (Colorfastness to Laundering: Accelerated Using a Launder-O-Meter) in a Launderometer model LEF (produced by SDL ATLAS). Twelve 5.08 × 5.08 cm samples of each tested fabric type were prepared using dripping methodology (above), with six replicated exposed to bleach in testing and six without exposure. Samples were placed in a canister containing 50 stainless steel balls and AATCC standard detergent solution (with or without bleach). The sealed canisters were placed in the launder-O-meter with hot water (49 °C) for 45 min. When the process finished, samples inside the canister were rinsed and air-dried for 48 h. The color was measured in the Konica Minolta Chroma Meter CR-5, utilizing the CIE L*a*b* color space. A general linear model was used to analyze the interactions between the independent variables of laundry, fabric type, heat, and time of heating with a four-way ANOVA and Tukey–Kramer test (*p* < 0.05) to determine significant differences between groups. One sample for each fabric/treatment was randomly selected and was taken to be analyzed with SEM (below) to visualize any change from the control samples. The response of prepared pigmented fabrics to heating was then compared, with samples previously subjected to heat testing. 

#### 2.4.3. Heat Testing

Testing samples were prepared following methods described for the laundry test (using the same number of samples), then heated to one of three common dryer temperatures (low = 50 °C, medium = 65 °C, and high = 80 °C) in a forced air oven to simulate a home dryer. Initial color readings were taken after samples dried using a Konica Minolta Chroma Meter CR-5 colorimeter using the CIE L*a*b* color space, using the ∆E calculation 2000. Color change was then again measured after 30 and 60 min exposure to heat, and samples were removed with tweezers and transported in baggies to prevent dirtying. Once the color was measured, two samples were randomly selected from each fabric/treatment and analyzed via SEM (below) to visualize any change that occurred due to the heat. A repeated measurement ANOVA with a mixed model was performed to determine the difference in ΔE and any interactions between the independent variables fabric, time, heat, and treatment, and a Tukey Kramer test was performed to classify possible interactions. 

#### 2.4.4. Qualitative Analysis: SEM

The purpose of SEM was to visualize the physical interaction between the pigment when carried in raw linseed oil and the fabrics and how this varied between treatment groups and the control. All tested samples (noted above) were dried for 48 h at room temperature, then were mounted on aluminum studs 5 mm in diameter using carbon adhesive tape. Samples were coated following Vega Gutierrez (2016), with gold-palladium in a Cressington Sputter Coater 108 Auto (Cressington Scientific Instruments, Inc, Cranberry Twp, PA, USA) for 35 s to allow the samples to generate a coating that decreased the electron charging and increased the contrast on the sample images. Samples were analyzed with a FEI QUANTA 600F environmental SEM (FEI Co., Hillsboro, OR, USA). The electron spot used was 2 A and 2 kV (high voltage).

## 3. Results

### 3.1. Influence of Fabric Type on Coloration of Samples

The color intensity of dyed fabrics was found to vary significantly (df = 619, F = 150.15, *p* < 0.001) depending on the type of fabric, with Tukey groupings showing that low-density cotton had the most pigmentation (∆E = 35.58 ± 1.73), followed by high-density cotton (33.21 ± 3.54), and low-density polyester (27.61 ± 4.22). Nylon, high-density polyester, high-density wool, and low-density wool were not found to differ significantly and showed lower coloration overall. 

### 3.2. Mechanical Testing

#### 3.2.1. Tensile Strength

No significant difference was seen in tensile strength between untreated fabric samples, fabric samples treated with linseed oil alone, and samples treated with pigmented oil (df = 2, F = 1.75, *p* = 0.1806). The fabric type was found to significantly affect the sample tensile strength (df = 6, F = 114.55, *p* < 0.0001), though no interaction was found between the interaction of fabric type and treatment, which would have indicated differences in strength changes for one or more of the fabrics due to the pigment (df = 12, F = 0.25, *p* = 0.9944). 

SEM analysis showed differences in fiber morphology after tensile testing between tested fabric varieties. High-density cotton showed flattened or curvy twisted fibers close to the break area, with hook-like ends at the ends of fibers (Figure S1). Low density cotton also showed curvy, twisted fibers close to break point, with split fiber ends (Figure S2). Nylon showed flattening at the area of breakage and wide separation of fibers, with oil accumulation visible on their surface (Figure S3). High-density polyester showed uneven broken fiber ends and minor flattening of broken ends (Figure S4). Low-density polyester in contrast showed an irregular twisted surface and wavy fibers in the failure area, with oil accumulation evident (Figure S5). High-density wool showed flattened and elongated fibers with a sharp angle on failure sites, showcasing the separation of the cuticle from the fiber core (Figure S6). Low-density wool showed similar characteristics to high-density wool, with clear damage from the test (Figure S7).

#### 3.2.2. Tear Strength

The tearing strength test showed a significant interaction between fabric type and treatment (df = 12, F = 9.76, *p* < 0.0001), with low density cotton showing a significant difference between untreated samples and samples treated with pigmented or non-pigmented linseed oil. Control samples tore at a significantly lower strength of 912.58 N compared to pigmented oil samples. Tukey groupings showed that the low-density wool with oil had the highest tear strength performance (5068.36 N), while high-density cotton treated with oil had the lowest performance (649.67 N) in this test.

High-density cotton showed clear flattened, broken ends with accumulated oil at the ends (Figure S8), while low-density cotton showed twisted sharp ends and wrinkled fibril bundles (Figure S9). High-density wool showed an even shape in the failure area with no loss of cuticle integrity, though a flattened core was present (Figure S10). Low-density wool showed cuticle loss and irregular surfaces, with breaking areas showing visible structural damage (Figure S11). Nylon showed un-flattened fibers with occasional bracket-like structures with accumulation of oil close to the break area on fiber ends, though most sections did not show this oil clumping (Figure S12). High-density polyester showed angled edges and flattened fibers, with oil accumulation and waviness seen in broken fiber ends (Figure S13). These was also seen in low-density polyester, which also showed twisting in the fibers (Figure S14).

### 3.3. Laundry and Heat Testing

Color change in response to laundry and heat processes was found to vary in response to testing conditions. The four-way ANOVA interaction between fabric type, laundry type (bleach/no bleach), temperature, and time was not found to be significant, nor were any three-way interactions. Significant two-way interactions included those between heating time and temperature (df = 4, f = 9.07, *p* = 0.0001), laundry type and temperature (df = 4, f = 7.66, *p* = 0.0005), heating time and temperature (df = 2, f = 9.07, *p* = 0.001), fabric type and temperature (df = 9, f = 1.88, *p* = 0.0349), and fabric type and laundry treatment (df = 8, f = 24.67, *p* <0.0001). Overall, these significant interactions suggested that fabrics responded differently to pigmentation, with bleach leading to discoloration and increased exposure to heat (either over time or at higher temperatures) leading to more intense color. Drying temperature seemed especially important, as it was present in all significant interactions, although its effects were mediated by other variables. 

**Figure 2:**
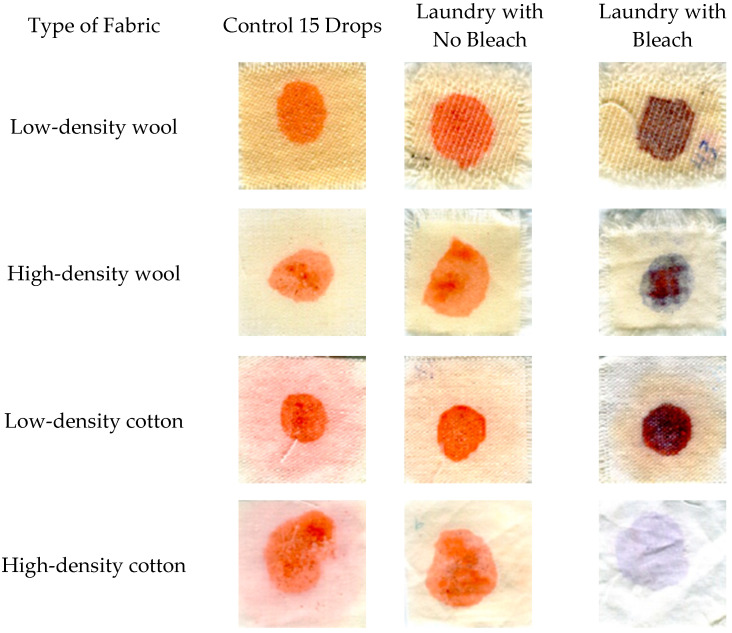

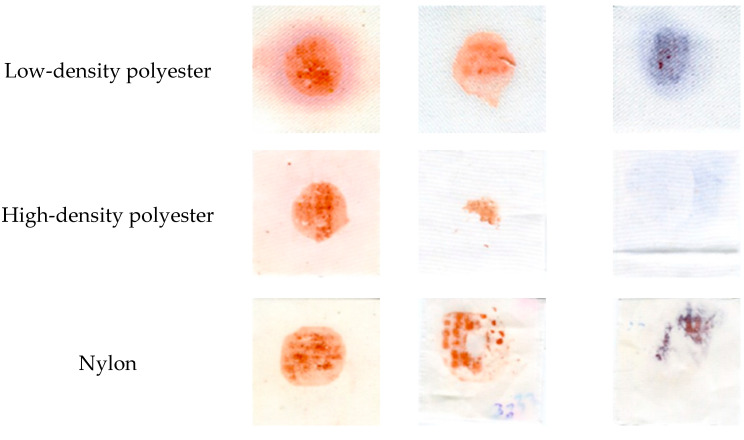
Differences in color and saturation between various fibers treated with 15 drops of dramada-pigmented oil across laundry without bleach, laundry with bleach, and the control (unwashed samples).

In general, the addition of bleach resulted in less color difference between pigmented samples and controls, although for low-density cotton, the addition of bleach resulted in a significantly higher ∆E (more color change). Polyester and nylon showed significant degradation of the pigments after laundry treatment, both with and without bleaching, as can be seen in Figure 1. However, in this case, the ∆E value does not tell the whole story. ∆E measures the total change in color, and, while in most cases in this study this change reflects the intensity of the red coloration, when bleach was applied on a number of fabrics, the hue of the pigmentation changed. In wool of both densities, the color changed from red to blue, and in cotton, the color changed from red to dark purple (Figure 2). Overall, bleach caused a more visible color change on natural fabrics over synthetic fibers. Synthetic fabrics followed a different pattern, presenting less color change after washing and drying. 

**Figure 3:**
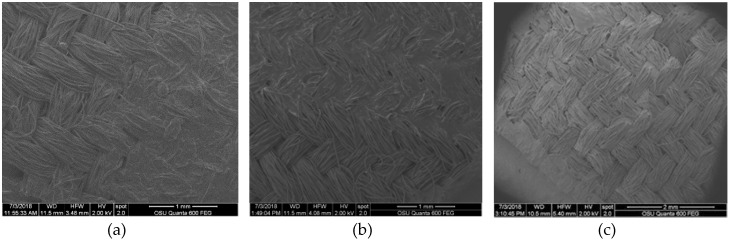
SEM images of a bleach/no bleach sample at the same temperature, showing degradation. (**a**) Low density polyester (control 15 drops); (**b**) low density polyester laundry without bleach; (**c**) low density polyester laundry with bleach.

In addition to the changes in color, as measured by ∆E, and the variation of this effect upon different fabrics, bleach seemed to have a profound effect on the applied pigment in oil, as was qualitatively seen in SEM. While fabrics did not appear degraded in SEM, pigments on samples showed evidence of breakdown. Figure 1 shows pigments looking different in the bleached sample compared to the unbleached sample on the same fabrics at the same drying temperature and time. The analysis of SEM images identified the loss of the pigment layer produced by the interaction of the fabric with the oil. As can be seen in Figure 3, the degradation of oil layers occurred regardless of whether bleach was used during laundry tests. When bleach was used, it severely altered the dye color. All cases showed a strong contrast in comparison with the initial coloration and/or a dramatic loss of the dyed area.

No dramada crystals were seen in cotton fabrics, with high density cotton showing a smooth covering of oil with no presence of crystals, which became porous after laundry treatment without bleach, and, in the presence of bleach, no oil was found on the samples (Figure S15). Low density cotton showed a less smooth surface of applied pigment with fissures on the outer layer, likely due to retention in the most superficial layer (Figure S16b). After washing with and without bleach, low density cotton showed retention of a thick layer of pigmented oil with an irregular porous shape (Figure S16). Nylon also showed no crystals in the pigmented oil layer, with an irregular surface that was actually smoother after laundry without bleach. In contrast, laundry with bleach resulted in stripping of much of the oil layer remaining only in spaces between yarns (Figure S17). 

High density wool also did not form visible crystals within the pigment layer, which formed a thick, even layer of oil that remained after laundry without bleach. However, laundry with bleach showed visible interstices and a porous layer (Figure S18). The low-density wool allowed for a higher degree of infiltration of the oil into the lower layer of the fabric than the high-density wool. Low density wool also showed a thick pigment layer covering the fibers, though interactions with inner layers of the wood fibers made it difficult to distinguish if a granular surface seen where pigment was directly applied was due to pigment/wool interaction or not. After laundry without bleach, the oil absorbance of the cotton became higher than wool or polyester, as seen through the interstices in the oil layer (indicating a dryer oil coat) (Figure S19c), with samples exposed to bleach also showing a reduction in oil and flaking along a porous surface. 

**Figure 4:**
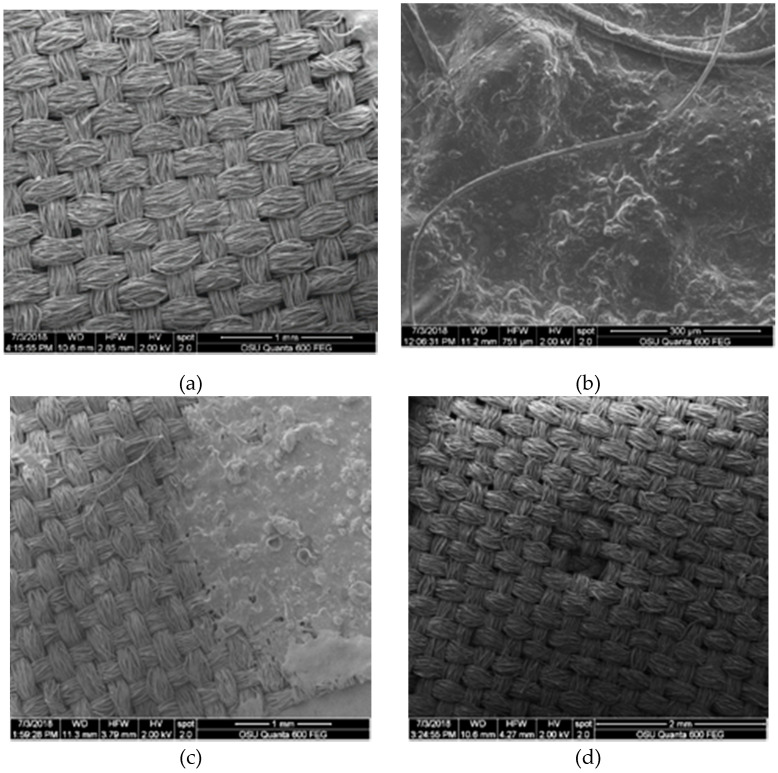
SEM images of high-density polyester samples: (**a**) control; (**b**) sample treated with 15 drops of pigmented oil, showing presence of dramada crystals; (**c**) pigmented sample after laundry treatment with no bleach, showing a somewhat smoothed surface; (**d**) pigmented sample after laundry treatment with bleach showing no visible oil layer.

**Figure 5:**
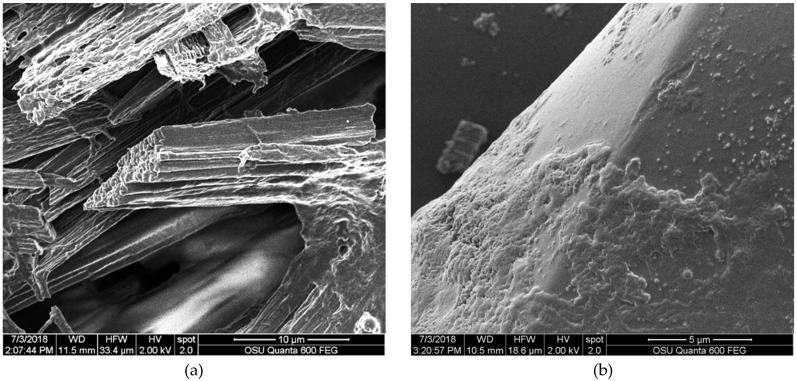
Distinctive features seen in polyester. (**a**) High-density polyester sample with a dramada crystal; (**b**) SEM image showing low-density polyester sample after the laundry test with bleach.

In contrast to other fabrics, polyester showed high concentrations of dramada crystals in below oil layers. High-density polyester showed a somewhat more even oil distribution, with a smoother surface seen after laundry with bleach and a lack of visible coating after laundry with bleach (Figure 4). Low density polyester also showed a reduced oil layer after laundry, along with several facture points. Oil also seemed to be degraded by detergent and bleach, forming cube-like structures between fibers and an overall porous surface (Figure 5). Low density polyester also showed a reduced oil layer after laundry, along with several facture points (Figure S20). 

### 3.4. Heat Testing

**Figure 6:**
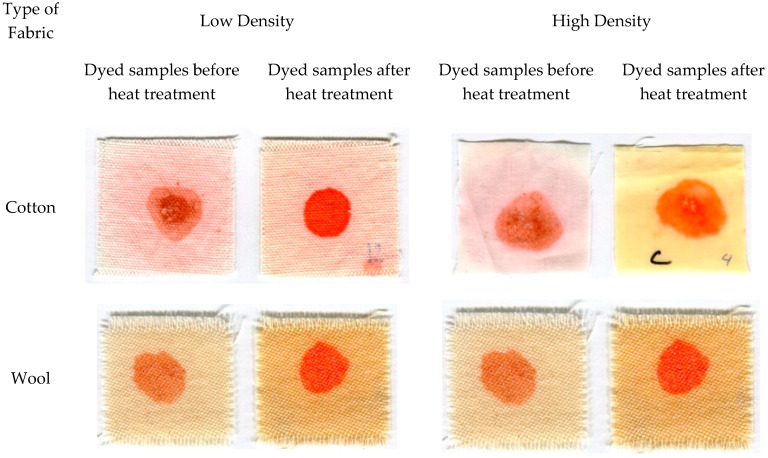

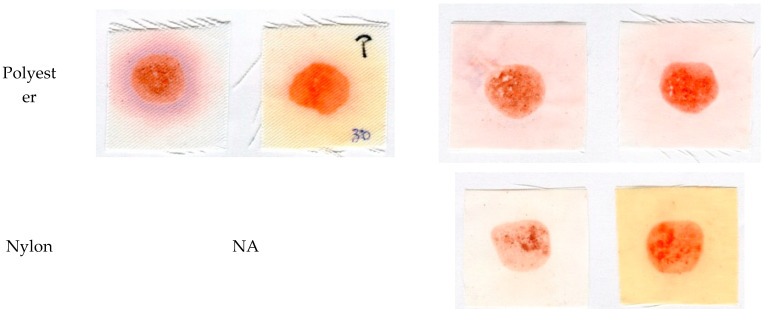
Fabrics dyed with pigmented oil before and after the heat treatment (30 min, 50 °C).

Significant variations of ∆E were seen between pigment treated fabrics exposed to drying conditions at either 50, 65, or 80 °C conditions for either 30 or 60 min. The four-way interaction between all these variables was not statistically significant (df = 12, F = 1.47, *p* = 0.1366), though two three-way interactions were found to be statistically significant. Fabric type and heating were found to influence color change after heating (df = 2, F = 7.41, *p* = 0.0008), with all fabrics having higher ∆E value after the heat treatment. In addition, the interaction between time, temperature, and treatment was significant (df = 12, F = 3.21, *p* = 0.0003), with an increase in ΔE after the heat process; a difference that can be seen in Figure 6. Tukey groups showed that high-density cotton and low-density polyester showed the highest (ΔE), while nylon showed the lowest (ΔE).

## 4. Discussion

The red pigment produced by *Scytalidium cuboideum*, carried in oil, was found to successfully dye a variety of fabric types without impact on tested mechanical properties, though wide variation in response to colorfastness treatments was seen. The color of the oil pigment varied based on fabric. These differences could be seen in the presence and morphology of the pigmented oil layer under SEM. 

The presence of the pigment in oil layer did not affect material strength or tensile strength. As natural dyes have historically been associated with weakening of fabric and decreases in tear strength due their need to use mordants for effective dying [26,27,28], this is a significant benefit for use of spalting fungal pigments. 

Pigmented low density cotton showed a significant difference in tear strength in comparison to untreated samples, but no significance was seen between pigmented oil and non-pigmented oil. This suggests that any differences might be due to the absorption of oil, which could have influenced slippage, leading to tearing. It is also possible that cellulose functional groups may have interacted with pigmented oil and formed a stronger bond; however, this would require further investigation. The fact that significance was only seen in this fabric type further suggests that this effect was not due to the pigment presence and instead suggests that factors, such as fabric thickness and diffusion, may influence dye absorption. Additionally, SEM showed that the pigmented oil did not stay in the internal structures of the high-density cotton but it did stay in the internal structures of low-density cotton, which had more space to absorb it. Both tensile strength and tear strength testing results mirrored those found by Hinsch [19], who reported no significant differences in strength values after application of *S. cuboideum* pigment in the DCM carrier.

While pigment presence was not seen to influence mechanical testing, clear differences in coloration of applied pigment were seen between fabric varieties with both cotton varieties and low-density polyester, showing significantly higher coloration than other fabric types (*p* < 0.001). It is likely that structural differences between fabric types are responsible for these differences in coloration. The more complex shapes created by natural fibers presented a different surface, leading to different interactions with the pigmented oil. A deep view with SEM showed that polyester fibers seemed to have the dye attached mostly in the outer areas of the sample. Polyester fabrics are normally hydrophobic materials; therefore, their affinity for oil-based compounds was supposed to be higher than that of cotton and wool (which are normally lipophobic materials). Previous research [1,19] did not show any relationship between this feature of the fabrics and their ability to be dyed; however, both studies found that polyester had a general affinity for natural dyes. This relationship still needs to be explored with other types of polyester and fabric blends.

While differences were seen between fabric varieties, density differences did not result in consistent patterns of pigment behavior. While it was speculated that the crystal structure contained in pigment from *S. cuboideum* could be entrapped differentially in fabrics of varying densities, SEM investigations showed that fabric density was also not related to crystal presence, with pigmented oil staying in the fibers without forming crystals. In most cases, a thin layer of oil formed on the surface and a marked accumulation was found in the intersections of each yarn. More crystals may have formed with time, as crystals from *S. cuboideum* have been shown to appear in oil after 60 days, potentially due to oxidation processes [29].

The natural fibers retained coloration better than the synthetics, with wool fabrics, in particular, showing stable color after laundering. This has been seen with other fungal dyes (although not from spalting fungi), specifically with *Talaromyces australis* (C.M. Visagie, N. Yilmaz & J.C. Frisvad) and *Penicillium murcianum* (C. Ramírez & A.T. Martínez) [30]. In most cases, laundry with detergent alone was sufficient to remove the oil layer from the applied pigment. It is likely that the detergent used in the laundry test affected the triester groups of the linseed oil through a complex formation with available -OH^−^ groups. However, complete removal was not seen on wool, and cotton samples though the coloration persisted, suggesting that the oil may influence pigment fixation, creating a mordant-like effect that has been described in other studies [31].

The pigmented oil layer degraded on exposure to bleach, likely through a saponification reaction. The strong alkalinity of bleach is also likely responsible for the color change seen in laundry samples. The pigment from *S. cuboideum* is known to turn blue under basic conditions, instead of its normal red coloration [32,33]. The mechanism behind this color change is under investigation but has not yet been described. It is possible that the variation in response to bleach seen may have been due to physical protection of pigment-functional groups pigment by the oil layer or by pigment binding to fabric surfaces. 

While bonding mechanisms of fungal pigment attachment to different fabrics were not tested, these likely impacted the differential responses seen in colorfastness. It is possible that hydroxy groups in cotton, and potentially amino acids in wool, may form hydrogen bonds with the methoxy and hydroxy groups present in the fungal pigment dramada. For the polyester fabric, it is likely that the aromatic π-electron system of the phthalic unit may be interacting with the π-electron system of dramada, providing a strong bond between the fabric and the pigmented oil. This interaction has already been reported with an anthraquinone present in natural dyes [1]. It is also interesting that. in previous research studies, polyester fabrics were successfully dyed with *S. cuboideum* when using DCM as a carrier [18] and later with raw linseed oil [25]. However, there is a need to further investigate which interaction causes the bonding between the fabric and the dye. 

Heating resulted in changes of fabric color, as it was present in all statistically significant interactions identified, with all fabrics brightening except nylon, which may have been due to low initial pigment uptake. Under SEM, a superficial layer could be seen on the dyed areas after heating. This layer was also seen in samples subjected to laundry testing and it appeared to be made of oil: it presented as a thin, compacted, smooth surface, and, in some cases, it showed interstices or pores. This layer was best seen in high-density wool and low-density cotton, which showed the best performance, as measured by ∆E after the heat test. The layer may have been formed due to evaporation, oxidation, or polymerization of the oil under the effect of the temperatures. The polymerization process of linseed oil is well known [34] and this process may have allowed for molecular stacking of the pigment into its crystal formation. Using high temperatures to accelerate the evaporation of the raw linseed oil has been previously reported in the preparation of oilcloths as a way to reduce drying time [35], although no oil layer, such as the one found in this research, has ever been reported. It is also notable that previous research has shown that temperatures higher than 70 °C have been shown to degrade the red color of dramada [29], although this effect was not seen during this research. It is possible that the oil provided some protection for the pigment. 

While this research showed that a range of fabrics could be effectively dyed using fungal pigment from *Scytalidium cuboideum*, this method of dying presented colorfastness challenges, especially for synthetic fibers. In addition, the texture of completed cloth may be an obstacle, as the oil does not polymerize to a dry layer quickly, making samples oily. However, treatment of cotton with linseed oil to create a waterproof layer, known as an oilcloth, was commonly done in the past [36] and may be a sustainable waterproofing option. Testing other cellulosic fibers, such as bamboo, rayon, or hemp, should also be investigated, as should the water repellence of the pigmented oil layer. In addition, new technology would likely need to be developed to allow for industrial processing of quantities of this pigmented oil. The economics of this product would require further research. In addition, natural oils have been researched for protection of exterior wood in service [37,38], which may be an additional use. 

The challenges seen using pigment from *S. cuboideum* in linseed oil may limit the ability of this technology to be adopted in the market. However, the use of spalting fungal pigments like that from *S. cuboideum* still have great potential for use in the textile industry, especially as they can be used without the need for mordants [18]. Other natural pigments, such as those from plant sources, have relied on mordants for their color stability [39,40,41,42], and the lack of mordants needed for fungal pigments represents a significant advantage. While it was hoped that linseed oil would provide a less toxic alternative to DCM, future work into identifying new solvents appropriate for use with dramada would be helpful, especially for use with synthetic fabrics. Recent work using pigment from *S. cuboideum* in inkjet printers may be a particularly effective method of textile dying with fungal pigments [43]. 

## 5. Conclusions

Red pigment from *Scytalidium cuboideum* carried in linseed oil was found to effectively dye a variety of fabric varieties, with natural fibers showing better coloration and colorfastness overall. Mechanical properties were found to be essentially unaffected by the application of pigment. Colorfastness was shown to vary among fabrics, with application of bleach associated with a color shift from red to blue of the applied pigment. Heating was found to result in the production of a bright red fabric, which may have potential as a sustainable waterproofing treatment. 

## Supplementary Materials

The following are available online at https://www.mdpi.com/2309-608X/6/2/53/s1, Figure S1: SEM images of high-density cotton exposed to tensile strength test; Figure S2: SEM images of low-density cotton exposed to tensile strength test; Figure S3: SEM images of nylon exposed to tensile strength test; Figure S4: SEM images of high-density polyester exposed to tensile strength test; Figure S5: SEM images of low-density polyester exposed to tensile strength test; Figure S6: SEM images of high-density wool exposed to tensile strength test; Figure S7: SEM images of low-density wool exposed to tensile strength test; Figure S8: SEM images of high-density cotton for tear strength test; Figure S9: SEM images of low-density cotton for tear strength test; Figure S10: SEM images of high-density wool for tear strength test; Figure S11: SEM images of low-density wool for tear strength test; Figure S12: SEM images of nylon for tear strength test; Figure S13: SEM images of high-density polyester for tear strength test; Figure S14: SEM images of low-density polyester for tear strength test; Figure S15: SEM images of high-density cotton samples from laundry test; Figure S16: SEM images of low-density cotton samples from laundry test; Figure S17: SEM images of nylon samples from laundry test; Figure S18: SEM images of high-density wool samples from laundry test; Figure S19: SEM images of low-density wool samples from laundry test; Figure S20: SEM images of low-density polyester samples.
