# Supplementary material for: Oil-Based Fungal Pigment from Scytalidium cuboideum as a Textile Dye
Source: J Fungi (Basel). 2020 Apr 22;6(2):53. doi: 10.3390/jof6020053 (PMC7345869; doi:10.3390/jof6020053)
Supplement: Supplementary file 1 [file jof-06-00053-s001.pdf]

# Supplementary Materials

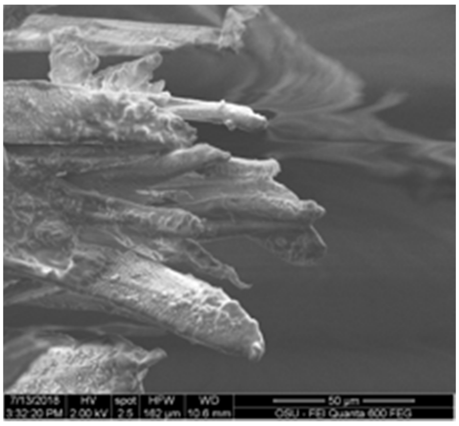


(a)

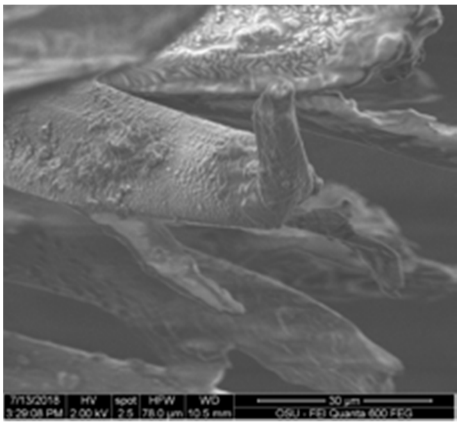


(b)

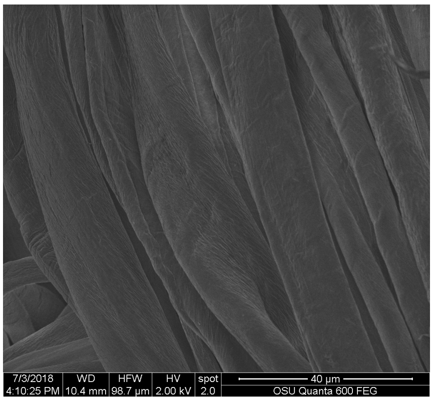


(c)

Figure S1. SEM images of high-density cotton exposed to tensile strength test. (a) Hook-like fiber ends. (b) Curvy-like ends in fibers. (c) Control sample.

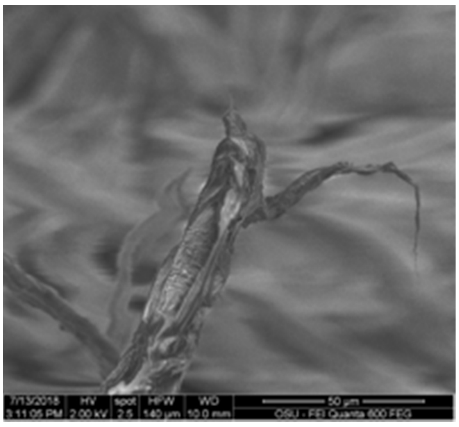

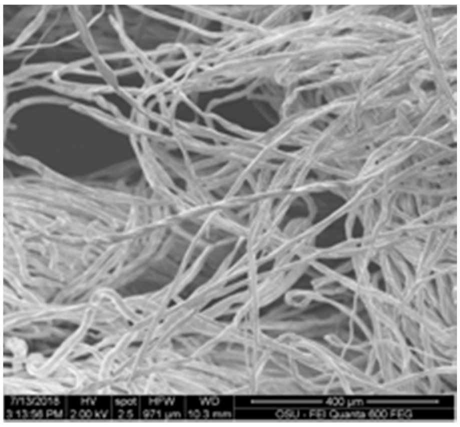

(a) (b)

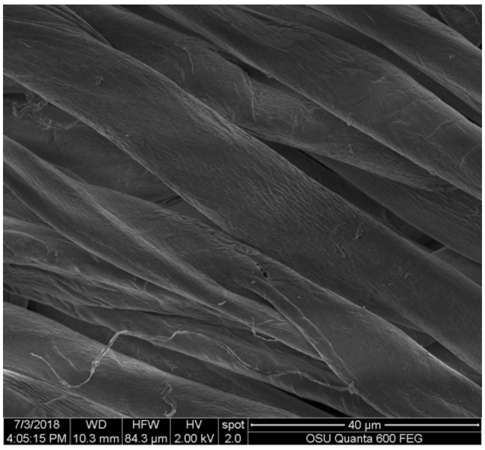

(c)

Figure S2. SEM images of low-density cotton exposed to tensile strength test. (a) Curvy end fibers. (b) Distribution of fibers after test. (c) Control sample.

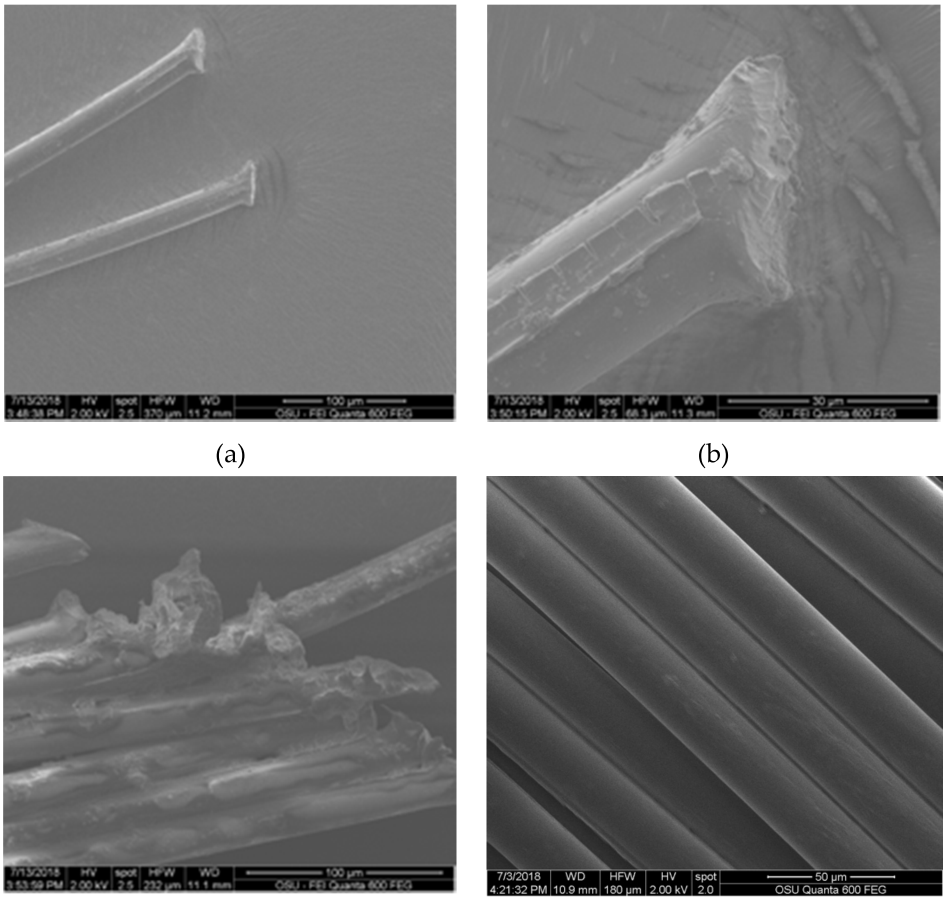

(a) (b) (c) (d)

Figure S3. SEM images of nylon exposed to tensile strength test. (a) Flattened end fibers on the breaking area. (b) Breaking area. (c) Separated yarns after test. (d) Control sample.

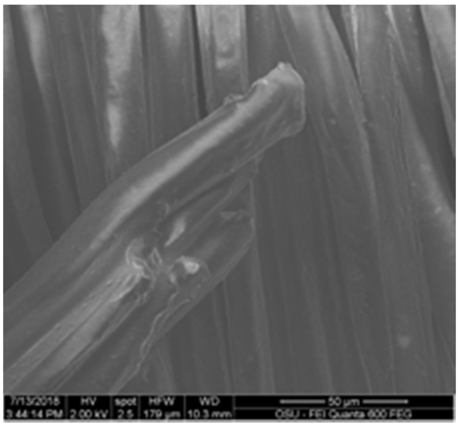

(a)

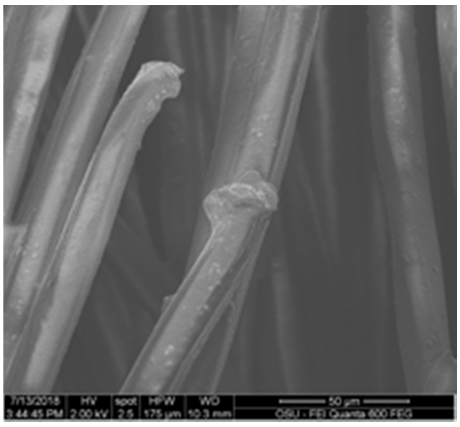

(b)

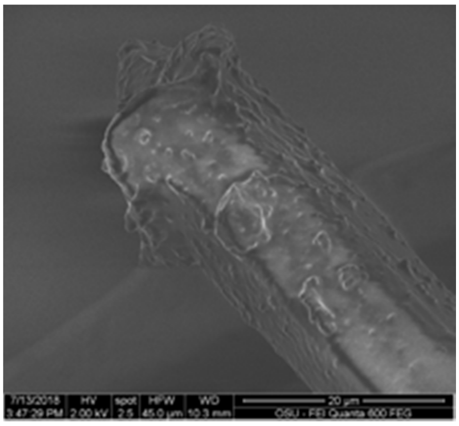

(c)

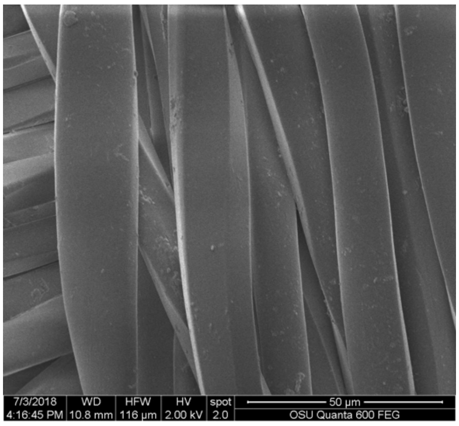


(d)

Figure S4. SEM images of high-density polyester exposed to tensile strength test. (a) Broken fibers. (b) Flattened area produced for the test. (c) Accumulation of the oil at the end of the fiber. (c) Control sample.

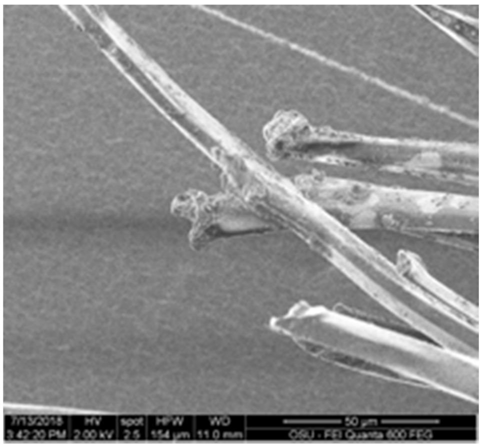


(a)

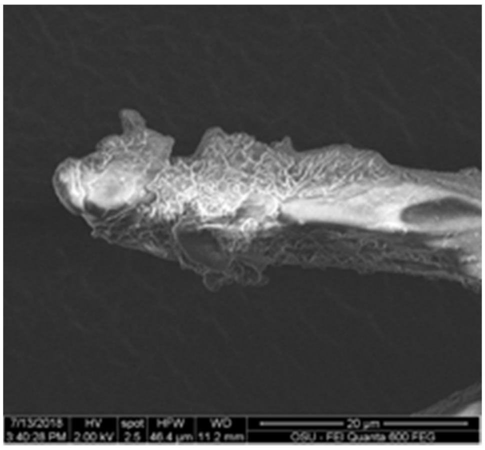


(b)

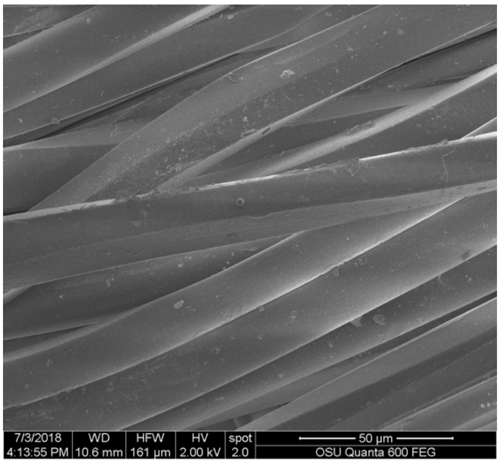


(c)

Figure S5. SEM images of Low-density polyester exposed to tensile strength test. (a) Flattened fiber ends. (b) Oil accumulated in the fiber ends. (c) Control sample.

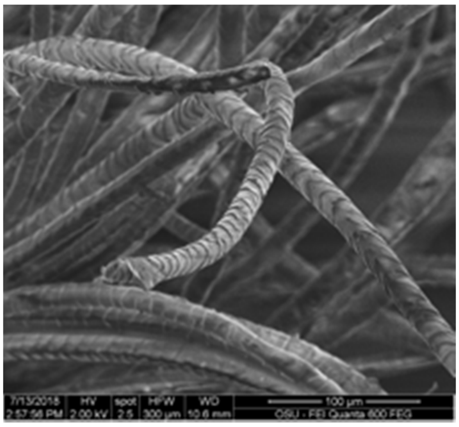


(a)

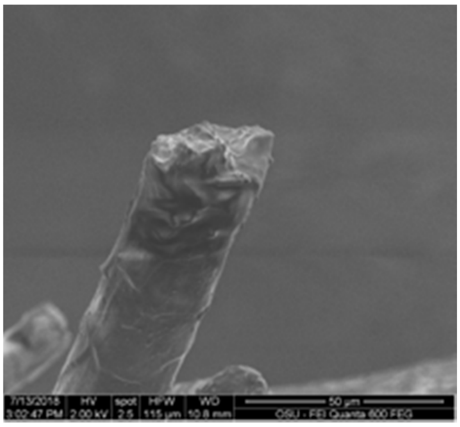


(b)

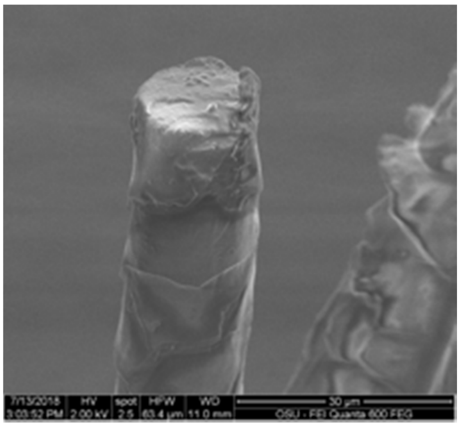


(c)

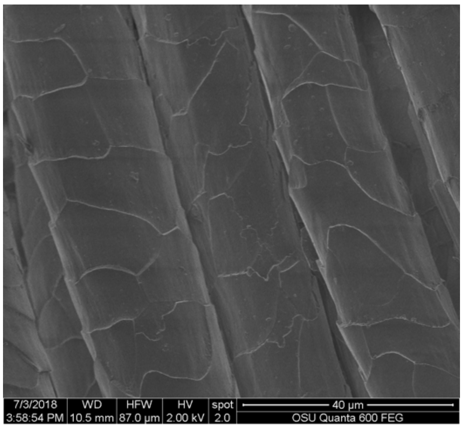


(d)

Figure S6. SEM images of high-density wool exposed to tensile strength test. (a) Distribution of fibers after test. (b) Loss of fiber cuticle. (c) Exposed fiber core. (d) Control sample.

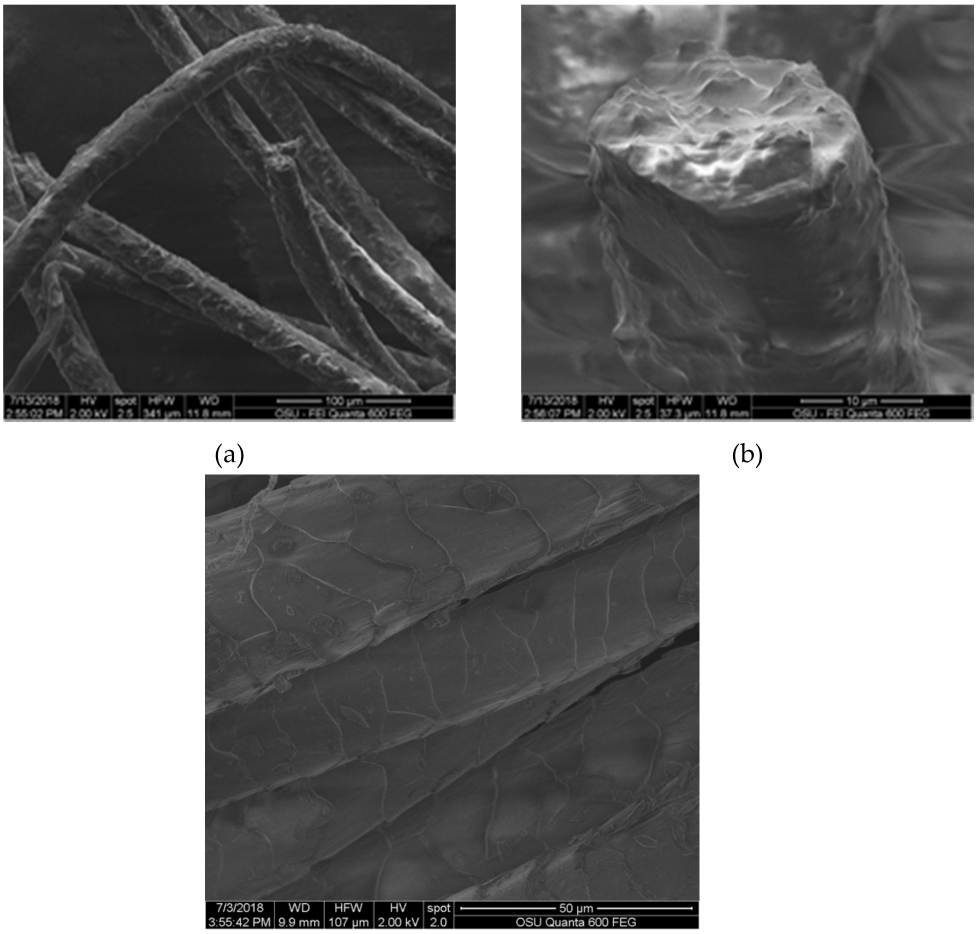


(a) (b) (c)

Figure S7. SEM images of low-density wool exposed to tensile strength test. (a) Wearing produced by the test in wool fibers. (b) Flattened areas produced in the breaking zone. (c) Control sample.

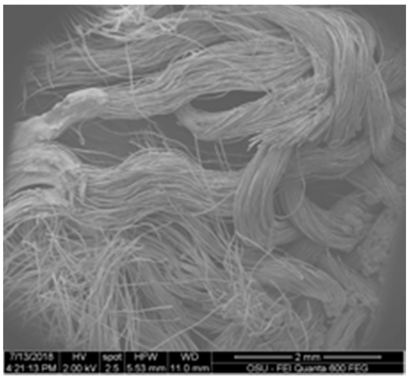


(a)

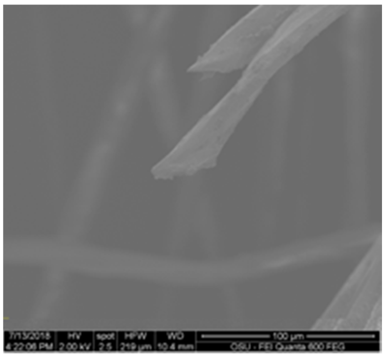


(b)

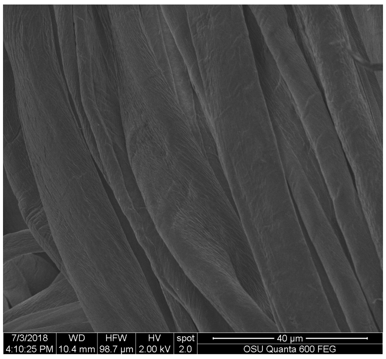


(c)

Figure S8. SEM images of high-density cotton for tear strength test. (a) Distribution of yarns after the test. (b) Pencil like shape after test. (c) Control sample. Fibers show flattened shape and superficial fibril loss though no fiber twisting.

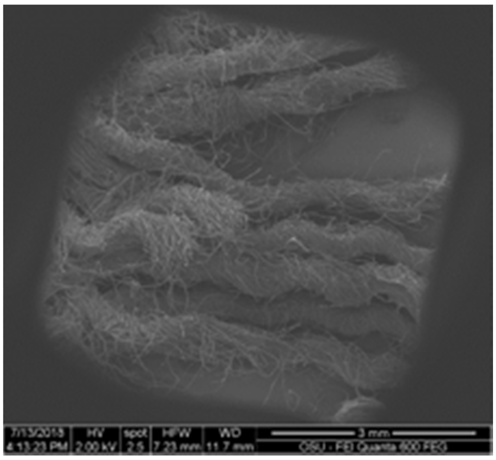


(a)

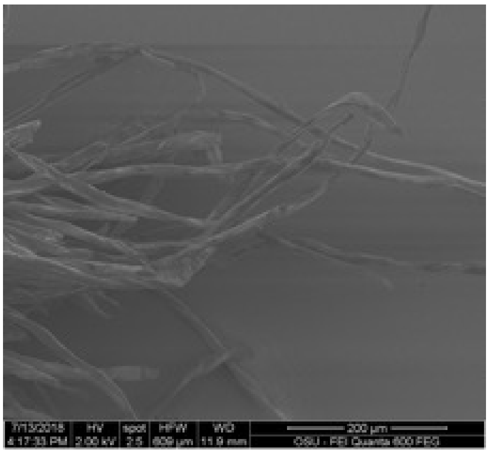


(b)

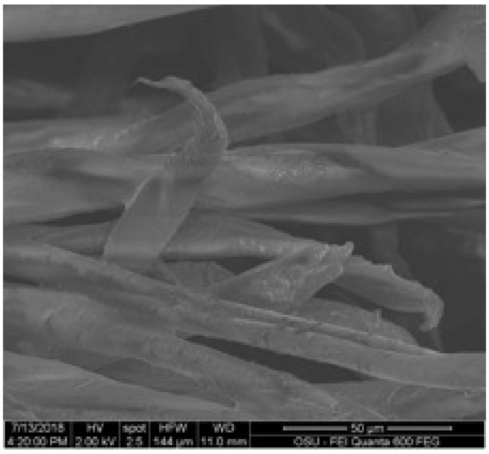


(c)

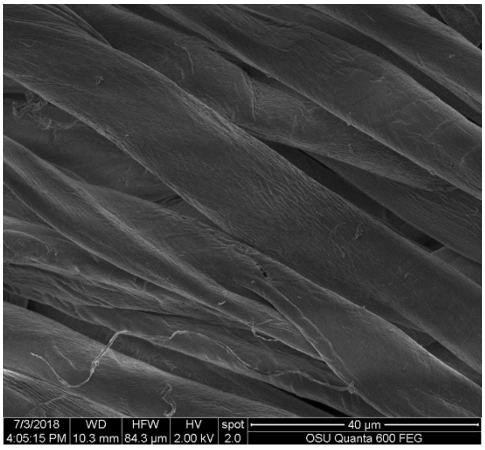


(d)

Figure S9. SEM images of low-density cotton for tear strength test. (a) Fiber rib ends (b) Fibril bundles. (c) Twisting effect in fabrics. (d) Control sample. Sharp ends, fiber twisting, and wrinkling with divisions of fiber bundles were seen.

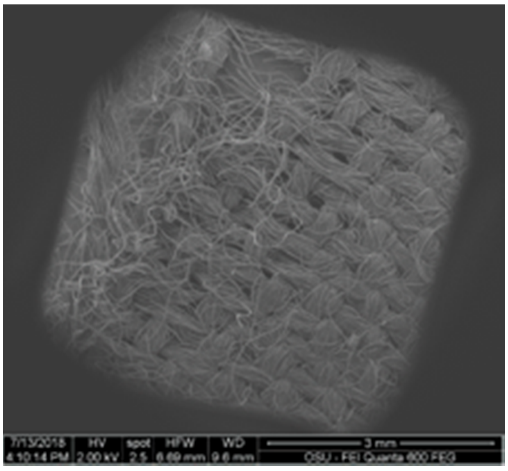


(a)

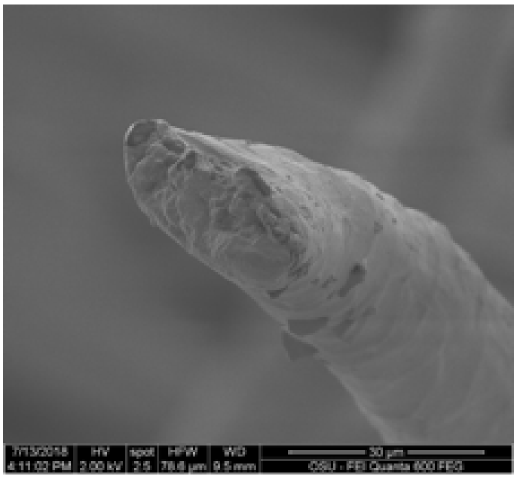


(b)

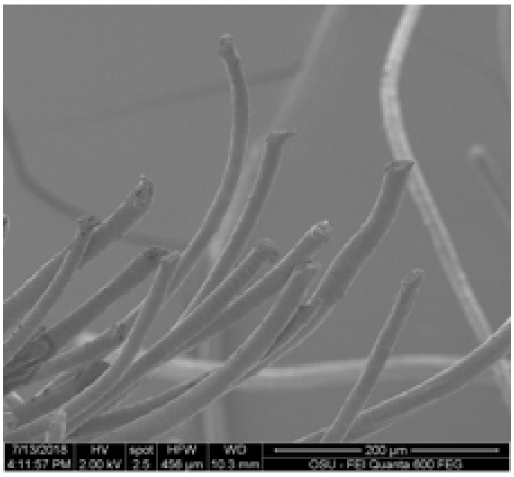


(c)

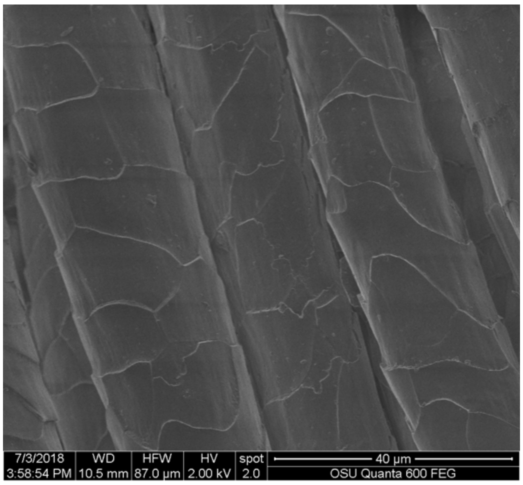


(d)

Figure S10. SEM images of high-density wool for tear strength test. (a) wearing in fabrics. (b) Fiber ends. (c) Failure area. (d) Control Sample. Samples showed even shape with flattening but no loss of cuticle integrity.

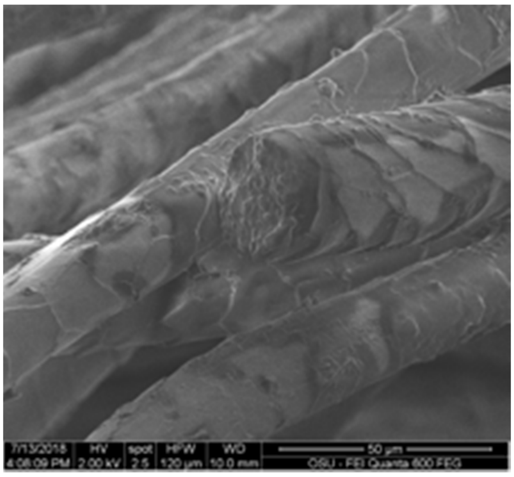


(a)

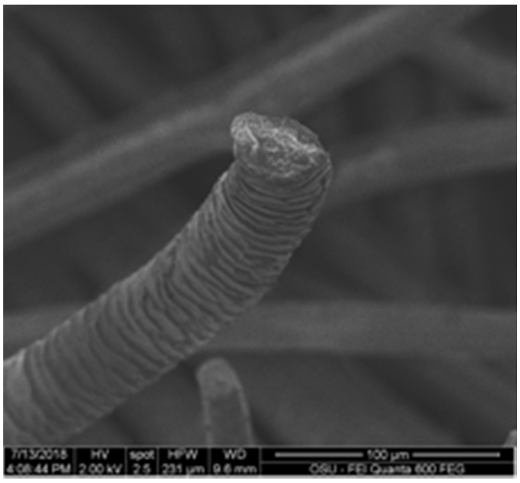


(b)

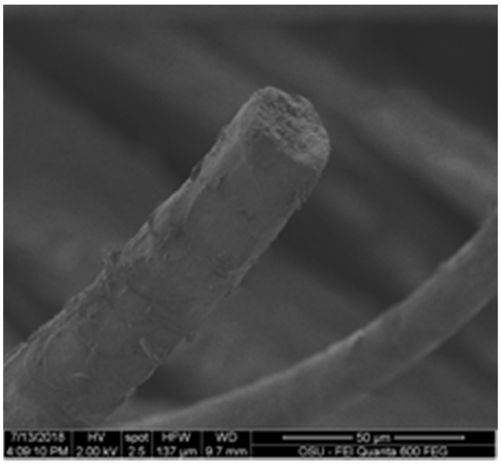


(c)

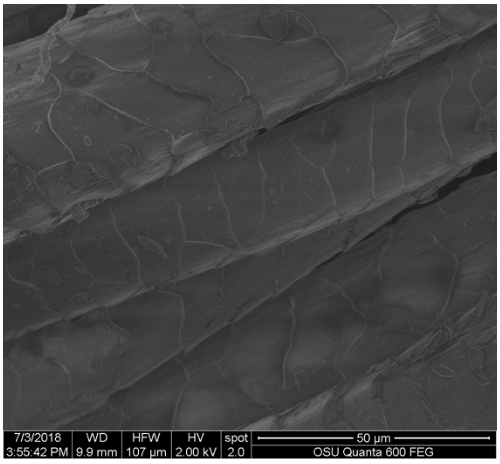


(c)

Figure S11. SEM images of low-density wool for tear strength test. (a) Fiber cut after tear testing. (b) Corrugated fiber after test. (c) Loss of cuticle. (d) Control sample. Irregular surfaces were seen at break area, with cuticle loss likely due to testing.

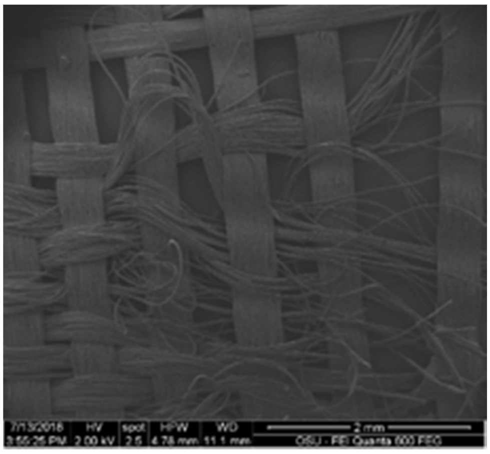


(a)

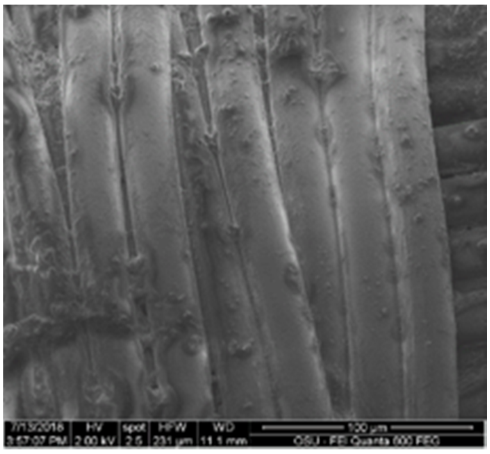


(b)

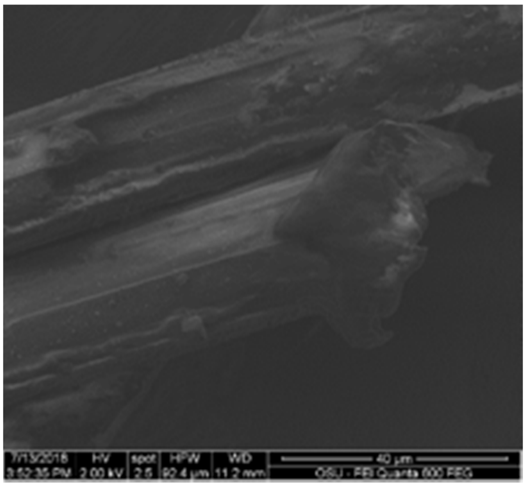


(c)

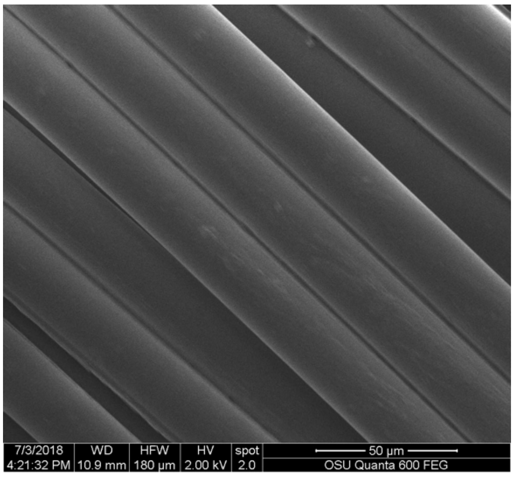


(d)

Figure S12. SEM images of nylon for tear strength test. (a) Distribution of yarns after the test. (b) Remaining oil along fibers. (c) Mushroom-like structure produced after test. (d) Control sample. Accumulation of oil was seen on fibers and close to the break area, though actual break area did not show oil distinct deposits.

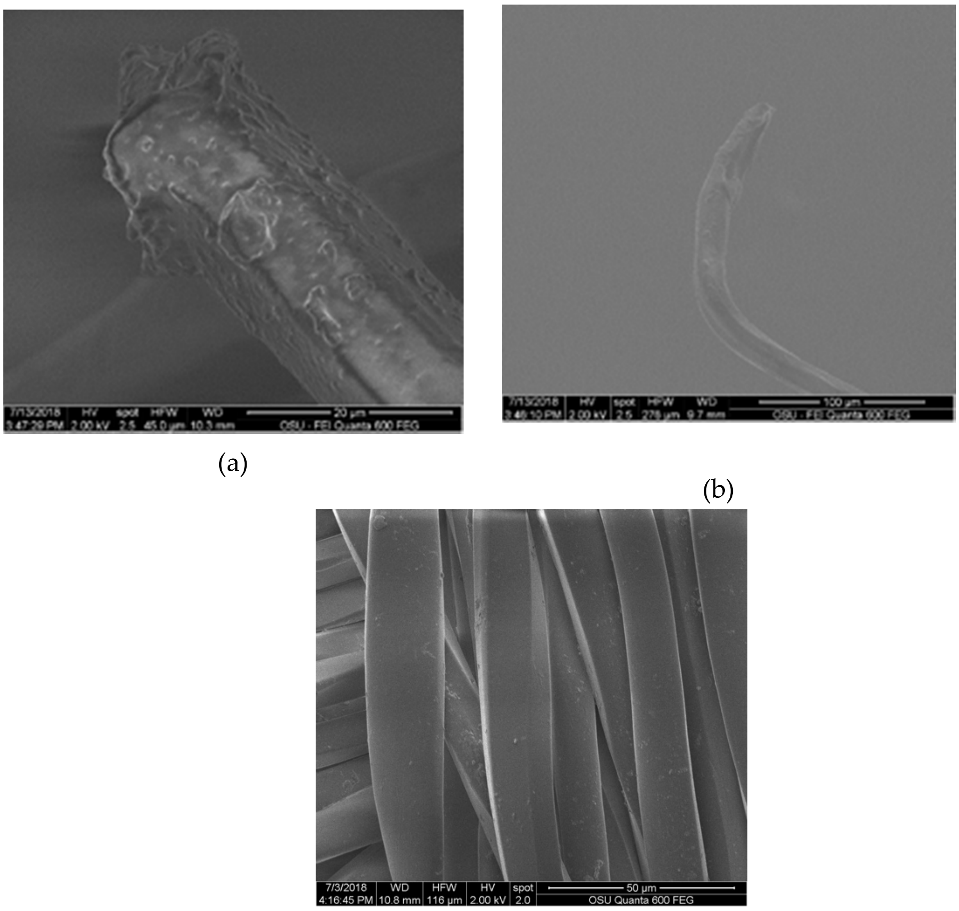


(a) (b) (c)

Figure S13. SEM images of high-density polyester for tear strength test. (a) Irregular surface at the end of the fiber (b) Flattened fiber ends. (c) Control sample.

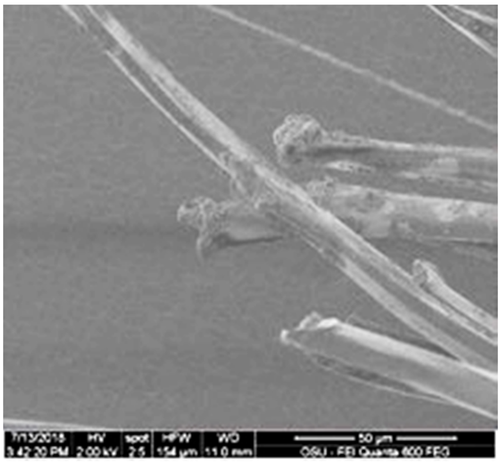


(a)

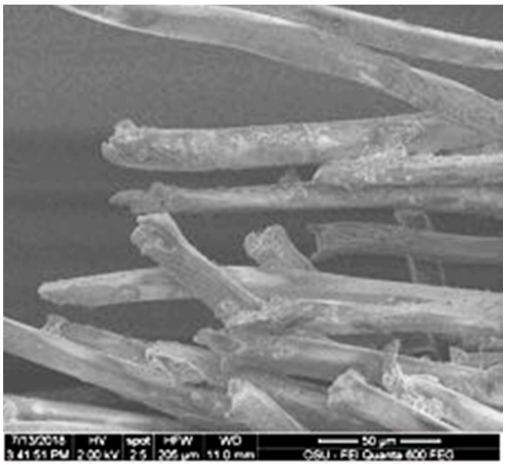


(b)

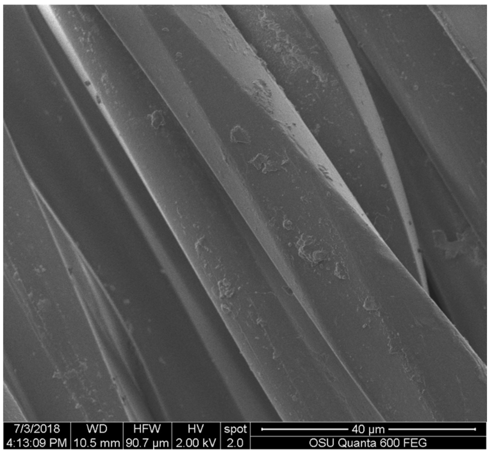


(c)

Figure S14. SEM images of low-density polyester for tear strength test. (a) Oil accumulation at the fiber end. (b) Twisted fiber in the breaking area. (c) Control sample. Angled ends were visible with no presence of oil.

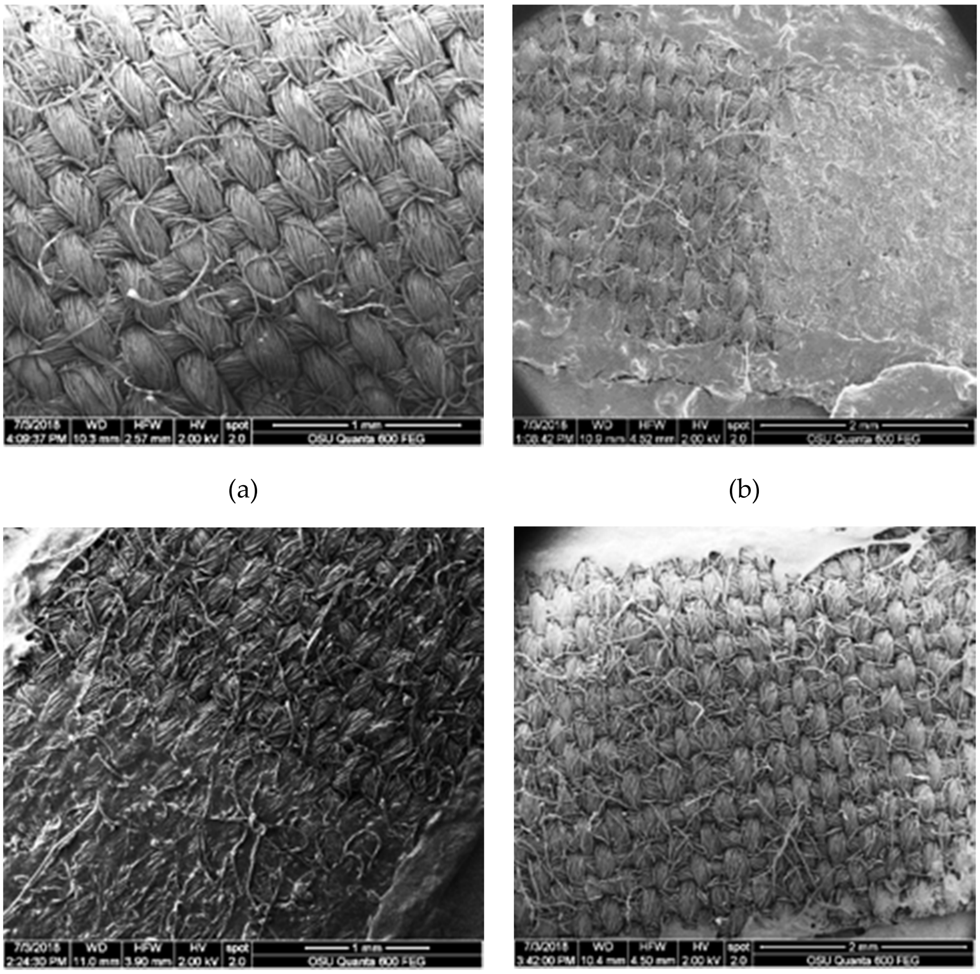

(a) (b)

(c) (d)

Figure S15. SEM images of high-density cotton samples from laundry test (a) Control; (b) Sample treated with 15 drops of pigmented oil; (c) Pigmented sample after laundry treatment with no bleach; (d) Pigmented sample after laundry treatment with bleach. No presence of crystal or segments of crystals were found on the oil layer, although in the intersection of the oil treatment and the diffusion areas (presence of thinnest oil layer) there was evidence of crystals forming. Some areas that presented the oil coat became porous after the laundry treatment, though laundry with bleach resulted in no visible presence of remaining oil.

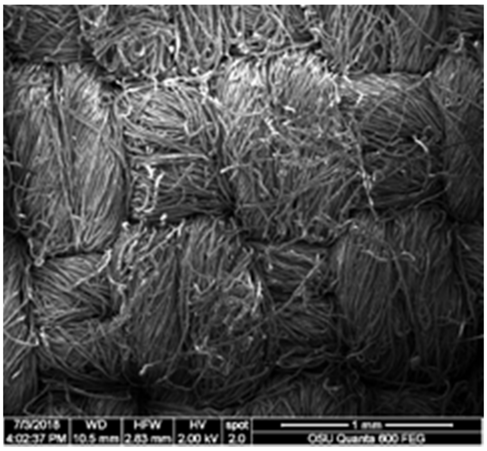

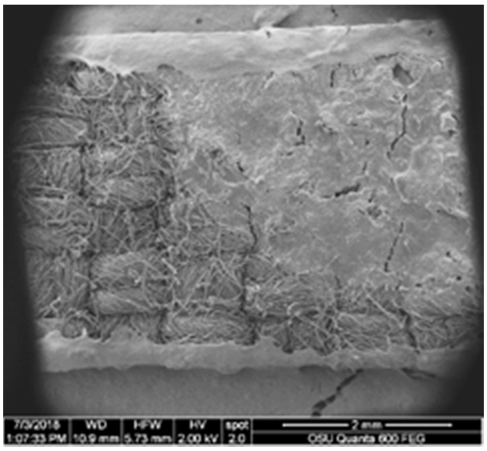

(a) (b)

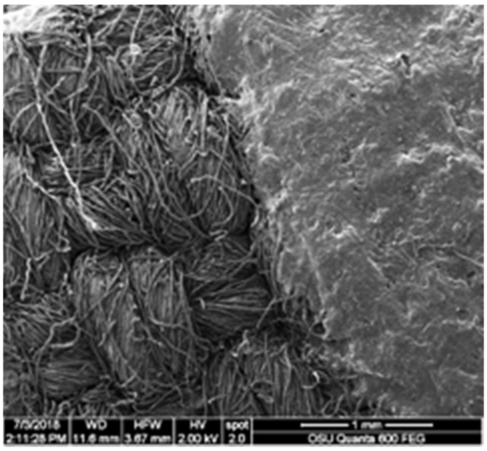

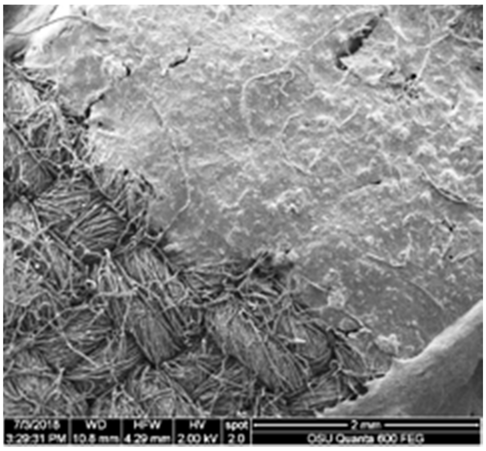

(c) (d)

Figure S16. SEM images of low-density cotton samples from laundry test (a) Control; (b) Sample treated with 15 drops of pigmented oil; (c) Pigmented sample after laundry treatment with no bleach; (d) Pigmented sample after laundry treatment with bleach. Images: Sarath Vega Gutierrez. Low density cotton showed a less smooth surface of applied pigment with fissures on the outer layer and a retention of a thick layer of pigmented oil with an irregular porous shape was retained after washing.

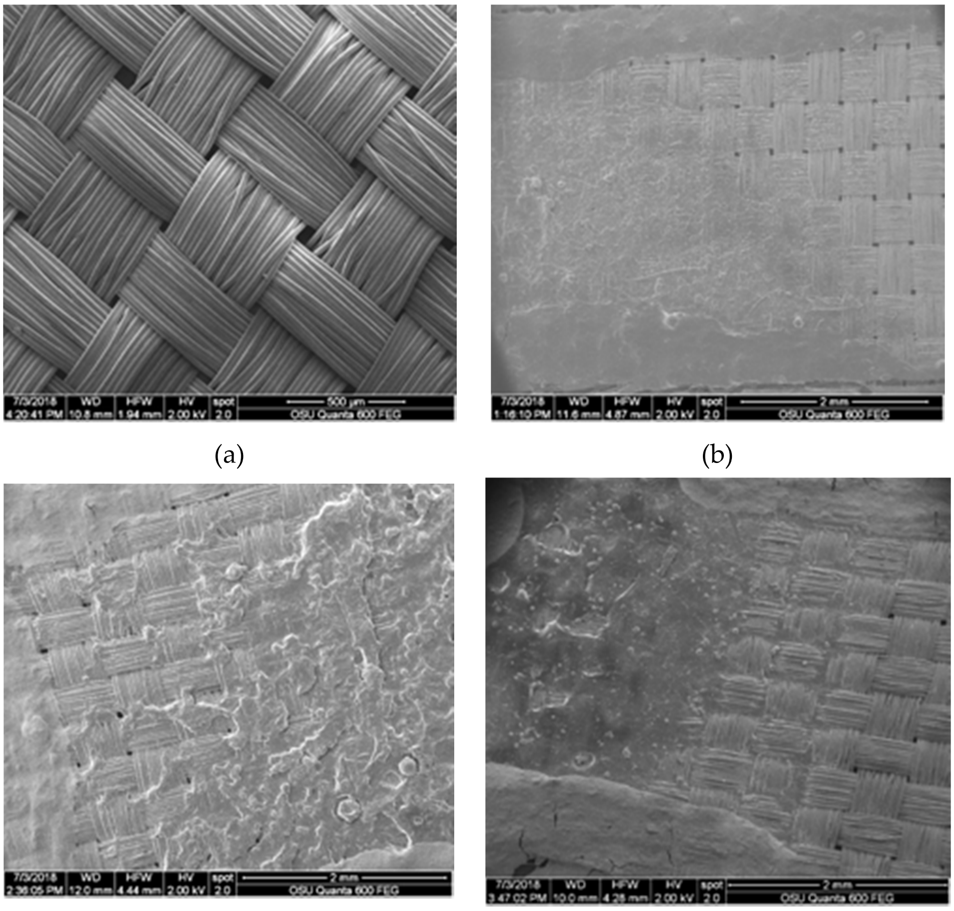

(a) (b)

(c) (d)

Figure S17. SEM images of nylon samples from laundry test (a) Control; (b) Sample treated with 15 drops of pigmented oil showing irregular surface; (c) Pigmented sample after laundry treatment with no bleach; (d) Pigmented sample after laundry treatment with bleach. Oil layer was smoother after laundry with bleach than without, particularly between yarns.

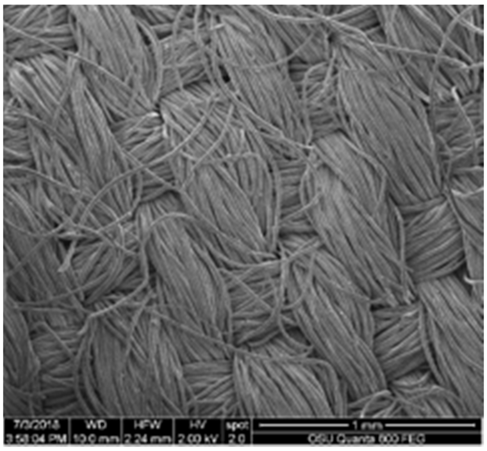


(a)

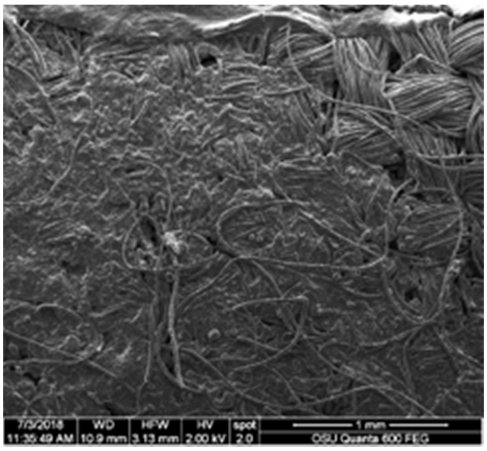


(b)

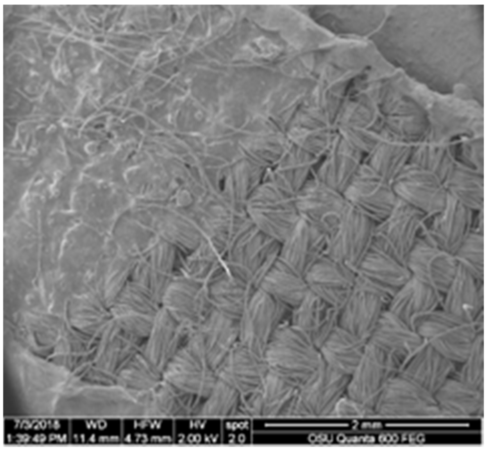


(c)

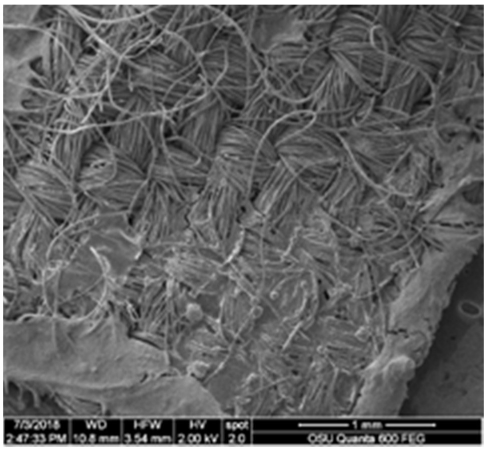


(d)

Figure S18. SEM images of high-density wool samples from laundry test (a) Control; (b) Sample treated with 15 drops of pigmented oil; (c) Pigmented sample after laundry treatment with no bleach showing thick even layer of oil; (d) Pigmented sample after laundry treatment with bleach showing visible interstices and porous layer.

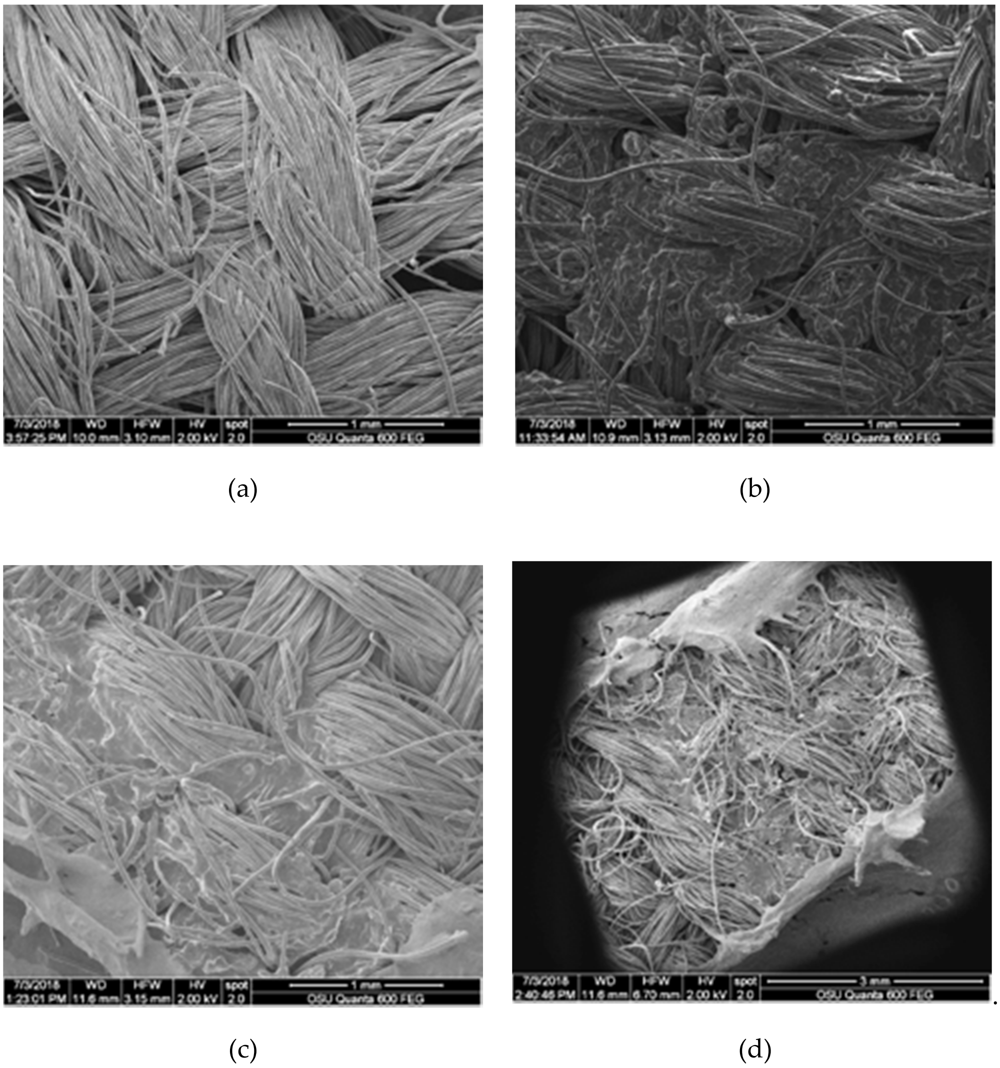


Figure S19. SEM images of low-density wool samples from laundry test (a) Control; (b) Sample treated with 15 drops of pigmented oil showing thick layer of pigmented oil; (c) Pigmented sample after laundry treatment with no bleach; (d) Pigmented sample after laundry treatment with bleach.

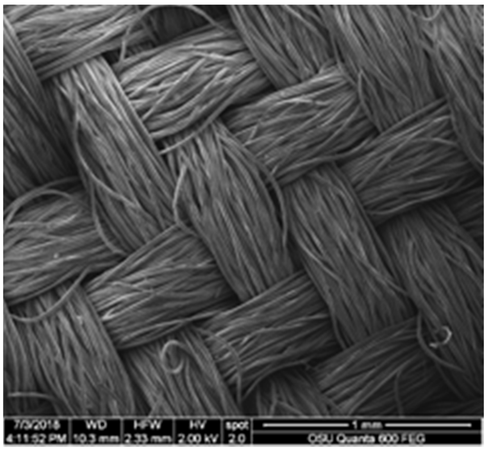


(a)

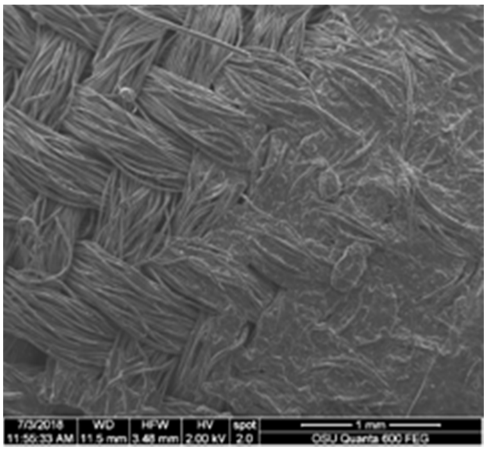


(b)

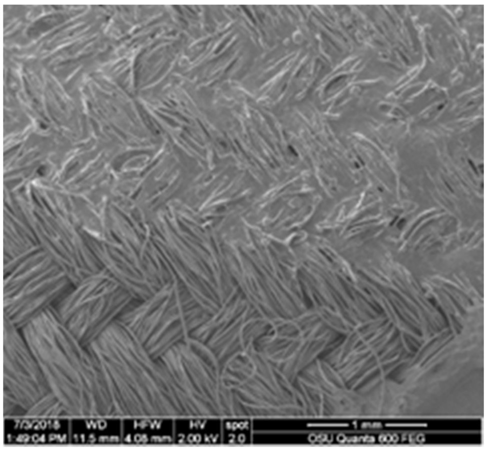


(c)

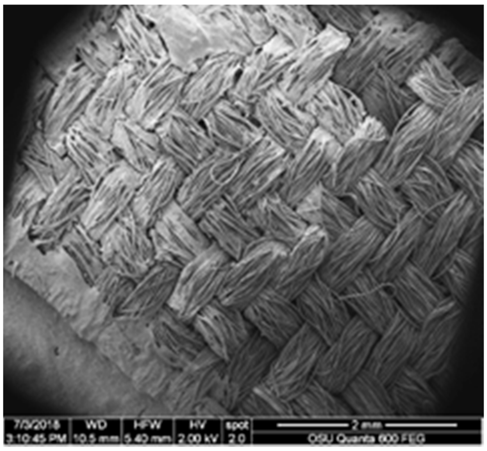


(d)

Figure S20. SEM images of low-density polyester samples (a) Control; (b) Sample treated with 15 drops of pigmented oil; (c) Pigmented sample after laundry treatment with no bleach showing reduced oil layer and fracture points; (d) Pigmented sample after laundry treatment with bleach, with formation of cube-like structure between fibers and overall porous surface.
